# Debromination of Hexabromocyclododecane by Anaerobic Consortium and Characterization of Functional Bacteria

**DOI:** 10.3389/fmicb.2018.01515

**Published:** 2018-07-10

**Authors:** Xingxing Peng, Dongyang Wei, Qiyuan Huang, Xiaoshan Jia

**Affiliations:** ^1^School of Environmental Science and Engineering, Sun Yat-sen University, Guangzhou, China; ^2^South China Institute of Environmental Sciences, Guangzhou, China

**Keywords:** hexabromocyclododecane, anaerobic reactor, debromination, bacterial variation, functional bacteria

## Abstract

A microbial consortium which can efficiently remove hexabromocyclododecane (HBCD) under anaerobic condition have been successfully enriched over 300 days. Under the optimal conditions, the degradation efficiency was 92.4% removal after treatment of 12 days with original addition of 500 μg/L HBCD, yielding 321.7 μg/L bromide in total as well. A typical debromination product, dibromocyclododecadiene (DBCD), was detected during the degradation process. The debromination profiles of three main HBCD diastereomers fitted well with first-order model (*R*^2^: 0.96–0.99), with the rate constants ranging from 1.3 × 10^-1^ to 1.9 × 10^-1^. The microbial community analysis by high throughput sequencing showed that the composition of the microbial communities varied dynamically with time and the population of functional bacteria increase sharply after enrichment. The population of *Bacteroidetes* increased from 5 to 47%. And some bacteria which are relatively minority in population at the beginning, such as *Azospira oryzae* (OTU2), *Microbacterium* (OTU13), and *Achromobacter insolitus* (OTU39) increased more than 22 times after enrichment (from 0.5 to 13%, 12%, and 11%, respectively). However, no reported dehalogenating bacteria were found after enrichment. And the contribution for debromination may come from new dehalogenating bacteria. All in all, the present study provided in-depth information on anaerobic microbial communities for HBCD removal by debromination.

## Introduction

Hexabromocyclododecane (HBCD) is one type of additive brominated flame retardants (BFRs) applied in various polystyrene resins and textiles to improve the flammability resistance or chemically bound to synthetic matrices such as plastics, textiles, electronic circuitry and other materials to prevent fires. Recently, it was also found in high tech devices, such as wind turbines and defense systems (www.bsef.com, 2016). Usually, it can be released during the industrial process or washed from waste products, then discharged into municipal wastewater treatment plants (WWTPs) or was washed from waste products and infiltrated various ecosystems. Up to now, the wide-spread existence of HBCD in the environment has drawn great attentions from many researchers due to the toxicity and persistence all over the world. It was reported in water (Morris et al., 2004), soil (Li et al., 2011), sediment (Kohler et al., 2008), plants (Li et al., 2011), animals (Law et al., 2006), humans (Eljarrat et al., 2009), and even indoor dust (Stapleton et al., 2008). Usually, the concentration of HBCD from μg/kg to mg/kg. Although HBCD is not acutely toxic, some research showed that its accumulation in the environment could cause adverse effects in human health, such as disruption of liver and thyroid hormone (Palace et al., 2008) and disorder of reproductions (Ema et al., 2008). Worse still, HBCD can accumulate along the food chain from the bottom to top, which is why the detection of HBCD serum concentrations in Norwegians is associated with the consumption of highly contaminated fish (Thomsen et al., 2008). Therefore, more studies for HBCD degradation are essential to eliminate its contamination in environment.

Conceptually, microbial degradation is to use living microorganisms to detoxify and degrade hazardous materials (Wang et al., 2014, 2015). It is generally considered to be an effective and safe way to remove contaminants from the environment, which has been widely applied in degrading pollutants such as pesticides (Chanika et al., 2011), plastic (Nakajima et al., 1999), additive (Wang and He, 2013), petroleum (Zhang et al., 2010), and surface-active agents (Fuchedzhieva et al., 2008). In our previous studies, biodegradation of TBBPA (another widely used BRF) has been successfully illuminated via analysis of bacterial consortium, isolation of pure culture, and deduction of co-metabolism pathway (Peng et al., 2012, 2013, 2014; Peng and Jia, 2013). To date, microorganisms have been reported to degrade HBCD both in aerobic and anaerobic conditions, such as *Pseudomonas* sp. Strain HB01, isolated from soil, can remove 81% of 1 mM γ-HBCD within 5 days of culture (Yamada et al., 2009). Under the anaerobic condition, a broken-down of technical HBCD mixture has been reported, (±)-a-HBCD exhibited an almost doubled half-life compared to (±)-β-HBCD and (±)-γ-HBCD (Davis et al., 2004; Gerecke et al., 2006). Usually, halogenated compounds can serve as electron acceptors in respiratory or co-metabolic processes, such as reduction of polychlorinated and polybrominated biphenyls (PCBs and PBBs) in anaerobic sediment enrichment cultures (Abraham et al., 2002). In addition, a small quantity of HBCD can be degraded in sediments, soils, and sewage sludge, while reductive dehalogenation (e.g., substitution of Br by a hydrogen atom) has been reported to be an important mechanism during biodegradation process under anaerobic condition. However, in most of the previous HBCD studies, the toxicology and the distribution of HBCD were emphasized, as well as the phenomenon of anaerobic degradation and degradation characteristics. Not enough attention has been paid to the acclimation and composition analysis of the microbial consortium. Hence, a more complete study including degradation characteristics, kinetics of HBCD stereoisomers, degrading pathway, and metabolites variation is in need.

**FIGURE 1:**
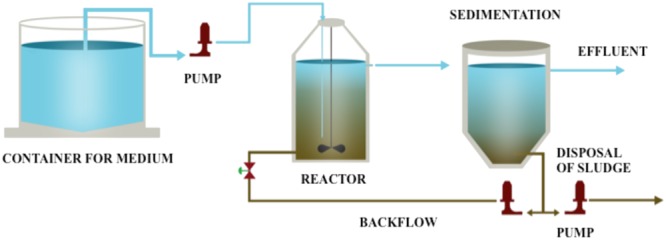
Schematic diagram of the reactor.

Overall, the aim of this study is to obtain and characterize the microorganisms that effectively biodegrade HBCD by enrichment. Considering the wastewater treatment facilities have processed wastewaters containing HBCD for several years, it is probable to possess microorganisms able to metabolize HBCD. In this study, laboratory reactor systems based on the conventional sewage sludge process were set up and operated for more than 300 days to develop a biological process for the debromination of HBCD (**Figure 1**). The optimal debrominated condition was analyzed by batch experiment. Subsequently, the degradation data was used to conduct fitted kinetic model. Moreover, the microbial composition was analyzed at different time points during the enrichment period (0–300 days at the interval of 60 days). Finally, the comparison of these samples at the phylum level and operational taxonomic unit (OTU) description were conducted. To our knowledge, a more comprehensive analysis of the bacterial community in the enriched bacterial consortium of HBCD was firstly unraveled in the present study, and a shifting pattern was discovered during the enriched time.

## Materials and Methods

### Chemicals

Hexabromocyclododecane (HBCD, 99% purity) was purchased from Sigma Chemical Co. (St. Louis, MO, United States), which was dissolved in acetone as stock solution (100,000 mg⋅L^-1^) before used. After filtration, it was rationed into the medium to obtain desired concentrations. All solvents (including acetone and methanol) used in this study were HPLC grade, which were purchased from Merck Company (Darmstadt, Germany). Other chemicals used for medium preparation were analytical grade and purchased from Sigma Chemical Co. (St. Louis, MO, United States). High quality water was obtained by using a Nanopure UV deionization system, Barnstead/Thermolyne Co. (Dubuque, IA, United States).

### Characterization of Sewage Sludge

The sewerage sludge was obtained from the anaerobic tank of wastewater treatment plant (WWTP) in Zhongkai industrial park of Huizhou, Guangdong province, China, which has been used to treat wastewater containing various BFR for a long time. Total solid (TS), volatile solid (VS), suspended solid (SS), and volatile suspended solid (VSS) analyses were based on the Standard Analytical Methods promulgated by the American Public Health Association (APHA, 1995). The crude fiber and crude protein were measured according to ISO 6865:2000 and ISO 1871: 2009, respectively. Crude fat content was determined using Soxhlet extraction method according to ISO 6492:1999. Carbon (C), hydrogen (H), and Nitrogen (N) were analyzed by an elemental analyser (PE2400). The general physical and chemical properties of the sewage sludge are showed in **Table 1**.

**Table 1:** General properties of the sewage sludge used in experiments.

Water content (%)	79.08 ± 1
TS (g/kg)	148.56 ± 0.5
VS (g/kg)	108.25 ± 0.2
SS (g/kg)	136.68 ± 0.6
VSS (g/kg)	102.86 ± 0.4
VSS/VS (%)	95.0 ± 2
Crude fiber (g/kg)	9.26 ± 0.02
Crude protein (g/kg)	62.74 ± 0.2
Crude fat (g/kg)	24.25 ± 0.1
C (%)	40.96 ± 0.2
H (%)	7.28 ± 0.02
N (%)	13.64 ± 0.5
C/N	3.0 ± 0.01

### Cultivation

The composition of the medium for the anaerobic reactor are as follows: NH_4_Cl 2,600 mg/L, MgCl_2_⋅7H_2_O 1054 mg/L, K_2_HPO_4_ 752 mg/L, CaCl_2_ 520 mg/L, and trace solution 1 mL/L. The carbon sources used in this experiment were as follows: NaHCO_3_ 800 mg/L, Na_3_C_6_H_5_O_7_⋅2H_2_O 680 mg/L, and C_6_H_12_O_6_ 1,000 mg/L. While the composition of the trace elements were listed as follows: NiCl_2_⋅7H_2_O 800 mg/L, FeCl_3_⋅6H_2_O 1,250 mg/L, ZnCl_2_ 130 mg/L, CoCl_3_⋅6H_2_O 110 mg/L, MnCl_2_⋅4H_2_O 220 mg/L, Na_2_BO_3_⋅10H_2_O 44 mg/L, (NH_4_)_6_Mo_7_O_24_⋅4H_2_O 80 mg/L, and CuCl_2_⋅2H_2_O 65 mg/L. The reactor was seeded with the anaerobic sludge incubated in the medium containing HBCD.

Initially, HBCD was added to the medium at concentration of 100 μg/L, and then the concentration was gradually increased from concentration of 200–500 μg/L. The pH of the mixture was adjusted to 7.0 before seeding. The anaerobic HBCD-utilizing sludge was enriched in a 3.0 L water-jacketed chemostat reactor at 30°C. The schematic overview used for the acclimation was shown in **Figure 1**. The reactor was running in continuous mode. Hydraulic retention time (HRT) was kept constant for 9 days, and the pH was maintained at 6.8–7.2 throughout the study.

### Batch Experiment Design

Several series of batch experiments were conducted with the successfully acclimated microbial consortia collected from the anaerobic reactor. In the batch experiments, 200 mL glass serum vials containing culture mixture were prepared for degradation study with three replicates. Samples were collected in the volume of 10 mL from batch reactor using glass syringe at each time point (0–12 days at the interval of 2 days) and filtered via 0.24 μm cellulose nitrate membrane. Among the 10 mL collections, 4 mL of which was used for TOC detection; the left was equally divided into three parts (2 mL/each), which were used for protein measurement, HBCD detection and bromide detection, respectively. Another series batch experiments were performed to select optimal culture conditions by comparison of different treatments. Different temperatures (20, 25, 30, 35, and 40°C), pH values (5, 6, 7, 8, and 9), HBCD concentrations (100, 500, 1,000, 5,000, and 10,000 μg/L), and carbon sources (sodium formate, sodium acetate, sodium propionate, sodium butyrate, and glucose) were test.

### Chemical Analysis

Total organic carbon (TOC) was determined using a Total Organic Carbon Analyser (Shimadzu TOC-VCPH, Kyoto, Japan). Because of low microbial content in 2 mL samples, the biomass content in the solution could not be measured directly by the VSS content. In this study, protein content was converted to VSS, which was analyzed according to the reported method (Zubkov et al., 1999). HBCD was analyzed by gas chromatography-mass spectrometry (GC-MS) with a fused silica column DB5-MS (30 m × 0.25 mm id, 0.25 μm dj) using He as the mobile phase. Three diastereomers of HBCD were determined by liquid chromatography-tandem mass spectrometry (LC-MS/MS) with the model of Agilent 6120. The analytical conditions were 10 mM ammonium acetate in water as phase A and 2% mobile phase B (methanol) at flow rate of 0.3 mL/min. The debrominated products were analyzed by ultra-performance liquid chromatography quadrupole time-of-flight mass spectrometry (UHPLC/Q-TOF-MS, Agilent 1290, Palo Alto, CA, United States; Bruker, Germany) using electrospray ionization (ESI) positive mode.

### Biodegradation Kinetics

Concentrations of individual diastereomers and ΣHBCD were normalized to the initial concentration vs. time. The biodegradation data were fitted to three decay models, i.e., zero-order (Eq. 1), first-order (Eq. 2) and second-order (Eq. 3).

Zero-order:

(1) $$\begin{array}{c} \frac{dc}{dt}=-k_{0}\Leftrightarrow C_{t}=C_{0}-k_{0}\cdot t \end{array}$$

First-order:

(2) $$\begin{array}{c} \frac{dc}{dt}=-k_{1}\cdot C\Leftrightarrow C_{t}=C_{0}\times e^{-kt} \end{array}$$

Second-order:

(3) $$\begin{array}{c} \frac{dc}{dt}=-k_{2}\cdot C^{2}\Leftrightarrow C_{t}/(1+C_{0}\cdot k_{2}\cdot t) \end{array}$$

Where, *C*_0_ is the initial concentration of substrate;

*t* is the degradation period in days;

*C*_t_ is the concentration of substrate at time *t*;

*k*_0_ is the degradation rate constant of zero-order;

*k*_1_ is the degradation rate constant of first-order;

*k*_2_ is the degradation rate constant of second-order.

The degradation half-lives (T1/2) were determined using the algorithm (Eq. 4) .

(4) $$\begin{array}{c} T_{½}=ln2/k \end{array}$$

### DNA Extraction, PCR Amplification, and High Throughput Sequencing

DNA samples were extracted from mixed culture at different time points (including 0, 60, 120, 180, 240, and 300 days) by using FastDNA-Spin Kit for Soil (MP Biomedicals, California, CA, United States) according to a modified protocol described previously (Urakawa et al., 2010). For high throughput sequencing, 16S rRNA gene of V4 region was amplified using specific primer of 515F and 806R with the barcode. All PCR reactions were carried out with Phusion^®^ High-Fidelity PCR Master Mix. Sequencing libraries were generated using TruSeq^®^ DNA PCR-Free Sample Preparation Kit (Illumina, United States) following manufacturer’s recommendations and index codes were added. The qualities of libraries were assessed on the Qubit@ 2.0 Fluorometer (Thermo Fisher Scientific) and Agilent Bioanalyzer 2100 system. At last, the libraries were sequenced on an Illumina HiSeq 2500 platform and 250 bp paired-end reads were generated. Paired-end reads were assigned to samples based on their unique barcode and truncated by cutting off the barcode and primer sequence. Paired-end reads were merged using FLASH. Quality filtering on the raw tags was performed to obtain the high-quality clean tags according to the QIIME (Bokulich et al., 2013). The tags were compared with the reference database using UCHIME algorithm to detect chimera sequences, and then the chimera sequences were removed (Edgar et al., 2011).

### Construction of 16S rRNA Gene Clone Libraries

For better parallel the bacterial shift of baseline (no enrichment, 0 day) and final enrichment (300 days), 16S clone libraries based on 200 clones were constructed. 16S rRNA genes sequences were amplified with the forward primer 27f: 5′-AGRGTTTGATCMTGGCTCAG-3′ and the reverse primer 1492r: 5′-GGYTACCTTGTTACGACTT-3′. The reactions were run on a Stratagene RoboCycler (Stratagene, La Jolla, CA, United States) under the following conditions: 5 min initial denaturing step at 95°C followed by 25 cycles of 30 s at 95°C, 30 s at 55°C for annealing and 2 min at 72°C for extension. A negative control (reagent only) was conducted during DNA extraction, as well as positive and negative controls were conducted during PCR process. All PCR products were purified using Qiaquick PCR purification kit (Qiagen, Valencia, CA, United States) and cloned into the PCR 4-TOPO Vector using the TOPO TA Cloning kit according to the manufacturer’s instructions (Version M, Invitrogen, Carlsbad, CA, United States). Two hundred positive clones of each sample were randomly selected by blue/white screening, then incubated in 100 μl LB broth overnight in 37°C. One microliter of cloned inserts was re-amplified using vector-specific primers (M13f-20 and M13r) to avoid co-amplification of *E. coli* host-cell DNA, as well as the PCR conditions were conducted as described above. The products suffered amplified rDNA restriction analysis by restriction enzymes (HhaI and HaeIII) separately under condition described by manufacturer (Invitrogen, Carlsbad, CA, United States), as well as the fragment pattern was determined by electrophoresis. Digestion pattern was analyzed using Gel-Pro Analyser Software version 6.0 (Media Cybernetics, Inc.) with 85 and 32 clones sequenced in the absence of repeated clones. Sequences that were less than 3% divergent were grouped into OTUs. Chimera sequences were removed using Ribosomal Database Project II (Maidak et al., 2001). Bellerophon (version 3) and excluded for further analysis. The sequences were compared to Gen-Bank entries using BLAST-n to obtain preliminary phylogenetic affiliations of the clones by the percentage of similarity.

### Statistical Analysis

Cluster analysis (CA) and UniFrac principal coordinate analysis (PCoA) are statistical techniques that sort observations into similar groups or sets, which were conducted using PAST software (Version 3.0). CA was based on taxonomy results and OTUs, whereas PCoA was based on RDP Classifier results, OTUs and UniFrac. It is a phylogeny-dependent method using phylogenetic information to compare the six samples.

## Results and Discussion

### Debromination of ΣHBCD and Determination of Optimal Conditions

**FIGURE 2:**
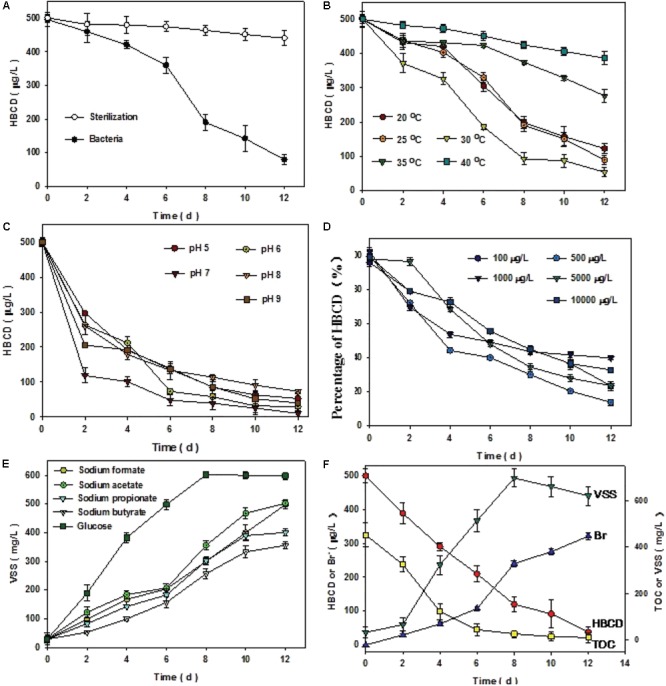
Comparison of sterilization and bacterial treatment **(A)**. Optimal conditions of microbial consortium degrading HBCD after enrichment of 300 days **(B–E)**. Variation of VSS, TOC, HBCD, and bromide ions under the optimal condition **(F)**. The left vertical coordinate represented that the concentration of HBCD or bromide ions (Br^-^), and the right coordinate represented the concentration of TOC or VSS. The initial concentration of HBCD and TOC were 500 μg⋅L^-1^ and 452 mg⋅L^-1^, respectively. The average of each gram of protein content in the mixed liquor is equal to 1.29 g biomass, i.e., VSS. Error bars represent the deviation of three replicates.

To elucidate the biotic or abiotic nature of ΣHBCD removal, sterile control was conducted (**Figure 2A**). The results of the treatment demonstrated that no significant removal of ΣHBCD was detected in the biomass-sterilizing microcosms. After 300 days, the results of experiments showed that compared with the sterilization batch, the microbe collected from anaerobic reactor degraded HBCD more effectively, with the HBCD concentration decreasing from 496 to 79 μg/L. Other parameters, including VSS, Br^-^, HBCD, and TOC were also measured to strengthen our understanding of the process. About 30°C, pH7, 500 μg/L initial concentration of HBCD, and using glucose as carbon resource were the best condition with degradation rate of 92.4% (**Figures 2B–E**).

Under the optimal condition, GC-MS analyses for ΣHBCD showed that the initial ΣHBCD masses added to the microcosms were 497 μg/L. Only relatively less biodegradation of ΣHBCD was observed in a lag phase of 6 h (from 497 to 421 μg/L). Thereafter, masses of ΣHBCD in test microcosms decreased sharply with the concentration from 421 to 69 μg/L, i.e., 86% of ΣHBCD had been degraded. Meanwhile, bromides were detected by using IC in the medium and its concentration increased to 321.7 μg/L after 12 days cultivation. The debromination rate is about 86% by mass balance. The dibromocyclododecadiene (DBCD, 322 m/z), a typical debromination product, was detected using UHPLC/Q-TOF-MS during reactive process (Supplementary Figure S1). Comparison of HBCD biodegradation and bromide generation, it could be deducted that bromide released from HBCD reduction by debromination process, which was consistent to the previous reports (Yamada et al., 2009). Transformation and correlation of VSS and TOC indicated that on the one hand, during 12 days cultivation, microbial growth experienced lag phase, log phase, and stationary phase, sequentially (**Figure 2F**; Fang and Jia, 1999); on the other hand, the biodegradation process with the generation of bromide was significantly negative correlated with the TOC analysis.

### Debromination of HBCD Diastereomers and Kinetics

**FIGURE 3:**
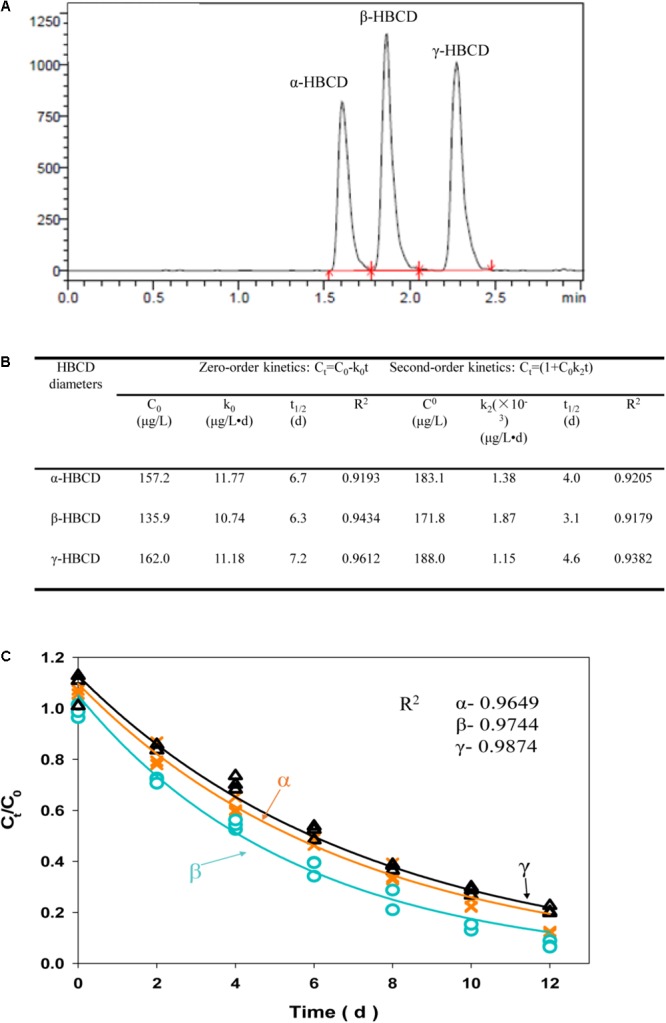
LC/MS/MS chromatograms of three HBCD diastereomers, including α-HBCD, β-HBCD, and γ-HBCD **(A)**. Degradation of individual HBCD diastereomers fitted the zero- and second-order model **(B)**. Degradation of individual HBCD diastereomers was fitted the first-order model **(C)**. The solid lines represent the predicted first-order decay curve deduced from the experimental data. The *R*^2^-value represents the coefficient for each HBCD diastereomers degradation model prediction

It was widely accepted that α-HBCD was the most dominant member among the three diastereomers accumulated in environment, part of which generated from selective biotransformation of β-, and γ-HBCD (Janák et al., 2005). In this study, three diastereomers, including α-HBCD, β-HBCD, and γ-HBCD, were successfully biodegraded by the acclimated microbial consortium in batch experiments (**Figure 3A**). In order to separately illustrate the biodegradation characteristics of the three diastereomers more accurately, three biodegradation kinetic models, including zero-order, first-order, and second-order, were applied to fit the debromination data of three diastereomers, (i.e., α-, β-, and γ-HBCD), respectively (**Figures 3B,C**). Determining from the HBCD biodegrading efficiency (*C*_t_/*C*_0_), it can be concluded that the biodegradation capability of the microbial consortium for the three diastereomers (α-, β-, and γ-HBCD), with the degradation efficiencies of 88.9, 92.6, and 79.1% after 12 days cultivations, respectively, were much higher than the previous reports that was only no more than 21% removal over 56-days incubation with active sludge (Davis et al., 2009; Harrad et al., 2009). No lag phase can be found in the biodegradation process. Biodegradation difference among the three diastereomers were found in the predicted *C*_t_/*C*_0_ curves, which followed the order of β-> γ-> α-, with consistence to the previous results discovered in the freshwater sediment mixtures (Morris et al., 2004), but was different to digester sludge with the order of β-> α-> γ- (Davis et al., 2009) and γ-> β-> α- (Gerecke et al., 2006), respectively. Compared to zero-order (*R*^2^: 0.92–0.96) and second-order model (*R*^2^: 0.92–0.94) (**Figure 3B**), the first-order model fitted biodegradation data of HBCD most with the *R*^2^-values ranging from 0.96 to 0.99. The half-lives for the three diastereomers were 4.3 days (α-HBCD), 3.6 days (β-HBCD), and 5.2 days (γ-HBCD), respectively, which were much shorter than that in the digester sludge described previously with only 50% removal of HBCD in treatment of approximately 15 days (Davis et al., 2009). The results were also not consistent with the previous observation that α-HBCD was debrominated slightly slower than β-HBCD and γ-HBCD (Gerecke et al., 2006).

### Bacterial Community and Diversity Analysis

**FIGURE 4:**
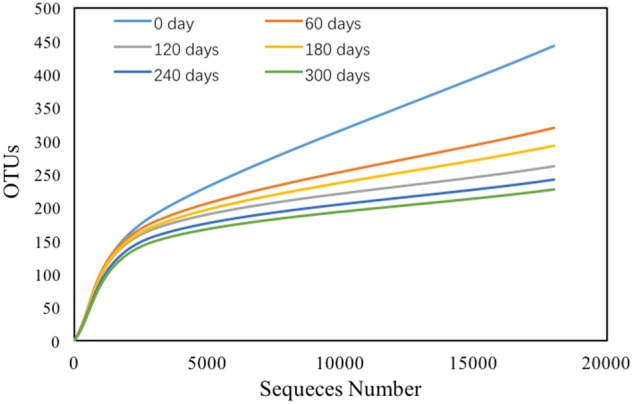
Rarefaction curves of six samples.

There were total 138,793 effective sequences obtained, and 2237 OTUs were observed at 97% similarity cutoff. The refraction curve of 97% cutoff similarity was shown in **Figure 4**, and the linearity of the rarefaction curve seemed to be smooth, suggesting that the sequencing depth was sufficient to describe patterns. The bacterial Goods coverage ranged from 0.992 to 0.995 (**Table 2**). All of these results demonstrated that the constructed library for each sample was fully satisfactory to characterize the bacterial communities. Considering Shannon-Wiener index (SWI) ranged from 1.213 to 3.542, the bacterial diversity declined with the enriched time increased. The trends of such index were almost in accordance with Ace and Chao’s index. The Simpson index also showed similar trend to the SWI.

**Table 2:** The α diversity of each sample.

Sample name	Time	OTUs	97% cutoff				
			Shannon	Chao1	Simpson	ACE	Goods_coverage
1	0	443	3.078	621.4	0.813	671.2	0.994
2	60	319	3.234	522.3	0.779	519.8	0.993
3	120	262	3.542	349.2	0.823	391.7	0.994
4	180	293	2.542	480.5	0.531	423.3	0.992
5	240	241	3.072	342.1	0.704	346.9	0.995
6	300	227	1.213	372.5	0.276	384.1	0.994

### Unifrac Clustering of Six Samples at Different Enriched Time

**FIGURE 5:**
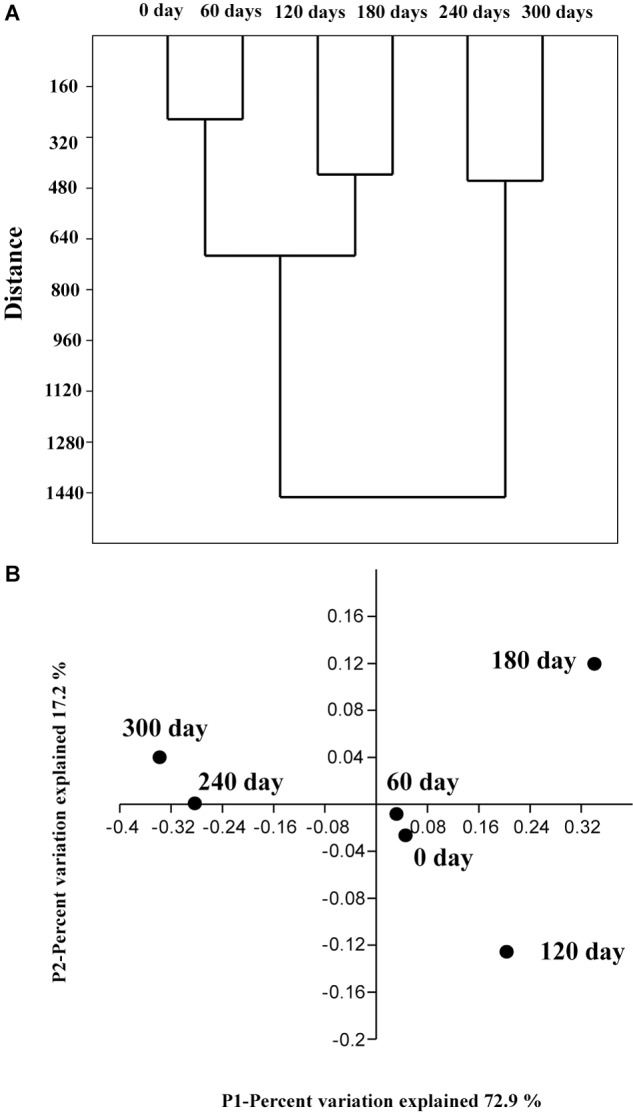
Cluster analysis based on the Bray–Curtis distance **(A)** at the genus level with a 3% cutoff. The genera were divided into three groups. The four-dimensional principal coordinate plot obtained using the genus results of samples collected from 6 enriched times in **(B).**

A clustering algorithm (based on Bray–Curtis distance) was applied to measure consistencies among various bacterial communities of the six enriched samples and to group similar samples at the genus level. As shown in **Figure 5A**, the bacterial communities in the six samples could be clustered into three groups: (1) Group 1 contains sample 1 (0 day) and sample 2 (60 days); (2) Group 2 contains sample 3 (120 days) and sample 4 (180 days); (3) Group 3 contains sample 5 (240 days), and sample 6 (300 days). It is obvious that the bacterial community changed with the enriched time.

In order to provide additional support for this analysis, a PCoA analysis was conducted on these validation data based on relative abundances at the genus level. The sample 1 (0 day), sample 2 (60 days), sample 3 (120 days), and sample 4 (180 days) were cluster together (samples 1 and 2 are much closer), whereas the other samples fall into another group. A hypothesis can be proposed that the bacterial shift with the enriched time is primarily because of the increase of HBCD concentration during the enrichment process, which was consistent with the previous CA analysis.

### Characterization of Bacterial Community

**FIGURE 6:**
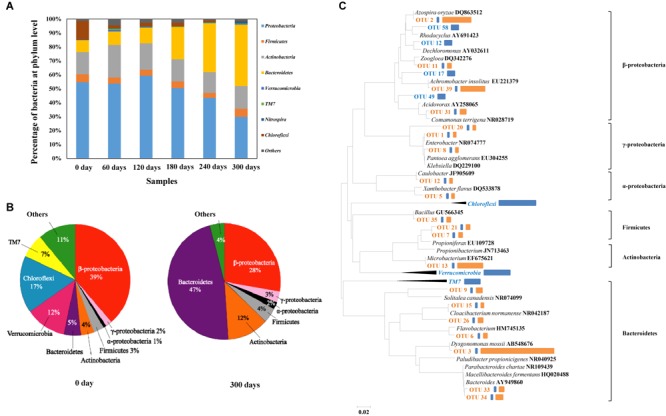
Abundances of phylums in the six enrichment bacterial consortium samples based on high throughput sequencing **(A)**. Distribution of clones in baseline control (i.e., 0 day) and final enrichment (i.e., 300 days) **(B)**. Phylogenetic tree showing the shift of the top 25 most abundant OTUs on which 16S rRNA gene sequences were identified, sequences of baseline control in blue, sequences of baseline and HBCD enrichment in orange **(C)**. Representatives of phylum *Chloroflexi, Verrucomicrobia*, and TM7 were used as outgroups for the analysis. Bars to the right of the dendrogram indicated the relative abundance of OTUs based on number of sequences. The blue represented the relative abundance in baseline control, while the orange represented the relative abundance in HBCD enrichment.

With the gradual increase of HBCD concentration in the process, i.e., from 10, 20–50 mg/L, microbial amount increased sharply (VSS: from 8.7 to 32 mg/L). The relative abundances of different phyla were analyzed, and in these six samples, *Proteobacteria* were the most abundant phylum, accounting for 30.5–59.3% of the total effective bacterial sequences (**Figure 6A**). The other dominant phyla were *Bacteroidetes* (8.6–44.9%, average 22.1%), *Actinobacteria* (14.8–23.6%, average 17.6%), *Firmicutes* (3.5–5.9%, average 4.8%), *Chloroflexi* (0.4–13.4%, average 3.7%), *Nitrospira* (0.07–1.8%, average 0.5%), *Verrucomicrobia* (0–1.2%, average 0.4%), *TM7* (0–0.7%, average 0.3%), and others (0.1–0.5%, average 0.2%). The results presented similar compositions at phylum level with another bacterial consortium degrading BFRs (Peng et al., 2012).

For clone library analysis, 128 clones from the library of baseline control culture and 56 clones from the library of HBCD microcosm were sequenced. Overall, as showed in the **Figure 6B**, the sequenced bacterial clones from baseline control culture refer to *Proteobacteria* (including α-, β-, and γ-*Proteobacteria* with the relative abundance of 2, 39, and 1%, respectively), *Firmicutes* (3%), *Actinobacteria* (4%), *Bacteroidetes* (5%), *Verrucomicrobia* (12%), *Chloroflexi* (17%), TM7 (7%), and unclassified group (11%), which presented similar compositions at phylum level with other sewerage sludge (Zhang et al., 2011). However, as showed in the **Figure 5B**, sequences from HBCD microcosm refer to *α*-*Proteobacteria* (2%), *β*-*Proteobacteria* (28%), *γ*-*Proteobacteria* (3%), *Firmicutes* (4%), *Actinobacteria* (12%), and *Bacteroidetes* (47%). It is worth mentioning that the pressure of HBCD greatly facilitated the growth of *Bacteroidetes* with the abundance from 5 to 47% in the process of acclimation, which may contain some functional microorganism degrading HBCD effectively. Moreover, *Actinobacteria* increased from the initial abundance of 4–12%. By comparison, *Firmicutes, α-proteobacteria*, and *β-proteobacteria* increased by a smaller extent, i.e., 1% more after HBCD treatment. However, the abundance of *TM7, Chloroflexi, Verrucomicrobia, β-proteobacteria*, and Unclassified group reduced in varying degrees. The previous report about the acclimation of microbial communities to mineralize the organic compounds indicated that some of enriched microorganism may be utilized to degrade the treated hazardous materials (Xiao and Roberts, 2013). Based on this, it can be speculated that the microbial enriched during the HBCD acclimated process may have a potential capability of degrading HBCD, just like *Pseudomonas*, which has been described previously (Yamada et al., 2009).

### Variation of the Bacterial Composition

Among the sequenced clones, 85 and 32 OTUs were identified from the baseline control culture and the HBCD microcosm based on the standard of OTUs (>97% identity), respectively (Kunin et al., 2010). Meanwhile, parts of typical OTUs with high relative abundance or radical change were selected for further research. Compositional and functional shifts in bacterial communities due to environmental factors, such as temperature (Gregory et al., 1997; Hao et al., 2010), have been widely observed in many natural and artificial ecosystems, such as soil, oceans (Gretchen et al., 2010), and reactor etc. In the present study, the HBCD treatment time-series variations in microbial diversity between baseline control culture and HBCD microcosm were analyzed and relative abundance of sequence, especially the top 25 OTUs with high abundance or radical shift were compared (**Figure 6C**). Overall, the abundances of *Bacteroidetes* (OTU3), *Actinobacteria* (OTU13), *β-proteobacteria* (OTU2 and OTU39) increased sharply. However, some microbe with abundant sequences in baseline control, such as *TM7, Chloroflexi, Verrucomicrobia* nearly decreased to zero after treatment with HBCD for different period. Especially OTU3, with 98% identity to *Dysgonomonas mossii* (AB548676), possessed the largest increase in relative abundance (from 0.5 to 34%) after 300-days incubation with gradually increased HBCD concentrations. Like the increase in abundance of OTU3, OTU2 (*Azospira oryzae*), OTU39 (*Achromobacter insolitus*), and OTU13 (*Microbacterium*) increased from 0.5 to 13%, 11%, and 12%, respectively, which also showed a potential preference to HBCD. Considering the tremendously increased abundance of particular clones in HBCD culture vs. the baseline control culture, it could be presumed that the taxa with radical increase in relative abundance may be important for anaerobic biodegradation of HBCD. Therein, the dominance of *Microbacterium* sp. in the HBCD microcosm supported the proposed role in the HBCD biodegradation process to debromination and further ring opening reaction (Håstein et al., 2006). In addition, a pure culture, identified as *Achromobacter* sp. by 16S rRNA gene sequence, has been isolated from the HBCD microcosm (unpublished data), which degraded HBCD effectively. Because HBCD degrading efficiency of the mixed culture is better than the pure culture of *Achromobacter* sp., other species obviously increased during the acclimated process, such as *Dysgonomonas* sp., *Azospira* sp., may also play important roles in HBCD degradation. It is worth mentioning that known bacteria for dehalogenation were not found after enrichment (Supplementary Table S1). New functional bacteria in reactive consortia may degrade HBCD by debromination.

## Conclusion

In the present study, bacterial consortium, effectively removing HBCD by debromination, were enriched by increased the contaminant concentration for over 300 days. Under the optimal conditions, bacterial consortium effectively degraded HBCD, and its three main HBCD diastereomers (α-, β-, and γ-HBCD) fitted first-order model well. The overall microbial community of samples collected from different enriched time had distinct patterns. New functional bacteria may degrade HBCD because no reported dehalogenating bacteria were found. Finally, compared to the sample collected from 0 day, bacterial community obviously varied with the acclimatization time, which was characterized by the special accumulation of *Bacteroidetes.* Over all, these results provided in-depth the anaerobic microbial communities exposed to HBCD.

## Author Contributions

XP and XJ conceived and designed the experiments. XP and DW did the bulk of data analysis. XP and QH performed the experiments. XP, DW, QH, and XJ wrote the paper. All authors have read and approved the final manuscript.

## Conflict of Interest Statement

The authors declare that the research was conducted in the absence of any commercial or financial relationships that could be construed as a potential conflict of interest.

## References

[B1] Abraham W. R. Nogales B. Golyshin P. N. Pieper D. H. Timmis K. N. (2002). Polychlorinated biphenyl-degrading microbial communities in soils and sediments. *Curr. Opin. Microbiol.* 5 246–253. 10.1016/S1369-5274(02)00323-5 12057677

[B2] APHA (1995). *Standard Methods for the Examination of Water and Wastewater.* 19th Edn. Washington, DC: American Public Health Association.

[B3] Bokulich N. A. Subramanian S. Faith J. J. Gevers D. Gordon J. I. Knight R. (2013). Quality-filtering vastly improves diversity estimates from Illuminaamplicon sequencing. *Nat. Methods* 10 57–59. 10.1038/nmeth.2276 23202435 PMC3531572

[B4] Chanika E. Georgiadou D. Soueref E. Karas P. Karanasios E. Tsiropoulos N. G. (2011). Isolation of soil bacteria able to hydrolyze both organophosphate and carbamate pesticides. *Bioresour. Technol.* 102 3184–3192. 10.1016/j.biortech.2010.10.145 21112209

[B5] Davis J. W. Gonisor S. J. Marty G. Friederich U. Ariano J. M. (2004). “Investigation of the biodegradation of [14C] hexabromocyclododecane in sludge, sediment, and soil,” in *Proceedings of the Third International Workshop on Brominated Flame Retardants* Toronto, ON 239.

[B6] Davis J. W. Gonsior S. J. Markham D. A. Friederich U. Hunziker R. W. Ariano J. M. (2009). Current-use brominated flame retardants in water, sediment, and fish from English lakes. *Environ. Sci. Technol.* 43 9077–9083. 10.1021/es902185u 19921842

[B7] Edgar R. C. Haas B. J. Clemente J. C. Quince Q. Knight R. (2011). UCHIME improves sensitivity and speed of chimera detection. *Bioinformatics* 27 2194–2200. 10.1093/bioinformatics/btr381 21700674 PMC3150044

[B8] Eljarrat E. Guerra P. Martínez E. Farré M. Alvarez J. G. López-Teijón M. (2009). Hexabromocyclododecane in human breast milk: levels and enantiomeric patterns. *Environ. Sci. Technol.* 43 1940–1946. 10.1021/es802919e 19368196

[B9] Ema M. Fujii S. Hirata-Koizumi M. Matsumoto M. (2008). Two-generation reproductive toxicity study of the flame retar-dant hexabromocyclododecane in rats. *Reprod. Toxicol.* 25 335–351. 10.1016/j.reprotox.2007.12.004 18262388

[B10] Fang H. H. P. Jia X. J. (1999). Formation of interim by-products in methanogenic degradation of butyrate. *Water Res.* 33 1791–1798. 10.1016/S0043-1354(98)00409-6

[B11] Fuchedzhieva N. Karakashev D. Angelidaki I. (2008). Anaerobic biodegradation of fluoranthene under methanogenic conditions in presence of surface-active compounds. *J. Hazard. Mater.* 153 123–127. 10.1016/j.jhazmat.2007.08.027 17869417

[B12] Gerecke A. C. Giger W. Hartmann P. C. Heeb N. V. Kohler H. P. Schmid P. (2006). Anaerobic degradation of brominated flame retardants in sewage sludge. *Chemosphere* 64 311–317. 10.1016/j.chemosphere.2005.12.016 16442150

[B13] Gretchen E. Hofmann J. P. Barry P. J. Edmunds R. D. Gates D. A. T. Hutchins D. A. (2010). The effect of ocean acidification on calcifying organisms in marine ecosystems: an organism-to-ecosystem perspective. *Annu. Rev. Ecol. Evol. Syst.* 41 127–147. 10.1146/annurev.ecolsys.110308.120227

[B14] Gregory P. Zogg D. R. David B. Ringelberg N. W. Macdonald K. S. Pregitzer D. C. W. (1997). Compositional and functional shifts in microbial communities due to soil warming. *Soil Sci. Soc. Am. J.* 61 475–481. 10.2136/sssaj1997.03615995006100020015x

[B15] Hao L. P. Lü F. He P. J. Li L. Shao L. M. (2010). Predominant contribution of syntrophic acetate oxidation to thermophilic methane formation at high acetate concentrations. *Environ. Sci. Technol.* 45 508–513. 10.1021/es102228v 21162559

[B16] Harrad S. Abdallah M. A. Rose N. L. Turner S. D. Davidson T. A. (2009). Current-use brominated flame retardants in water, sediment, and fish from English lakes. *Environ. Sci. Technol.* 43 9077–9083. 10.1021/es902185u 19921842

[B17] Håstein T. Hjeltnes B. Lillehaug A. Utne S. J. Berntssen M. Lundebye A. K. (2006). Food safety hazards that occur during the production stage: challenges for fish farming and the fishing industry. *Rev. Sci. Tech.* 25 607–625. 17094701

[B18] Janák K. Covaci A. Voorspoels S. Becher G. (2005). Hexabromocyclododecane in marine species from the Western Scheldt Estuary: diastereoisomer-and enantiomer-specific accumulation. *Environ. Sci. Technol.* 39 1987–1994. 10.1021/es0484909 15871228

[B19] Kohler M. Zennegg M. Bogdal C. Gerecke A. C. Schmid P. Heeb N. V. (2008). Temporal trends, congener patterns, and sources of octa-, nona-, and decabromodiphenyl ethers (PBDE) and hexabromocyclododecanes (HBCD) in Swiss lake sediments. *Environ. Sci. Technol.* 42 6378–6384. 10.1021/es702586r 18800504

[B20] Kunin V. Engelbrektson A. Ochman H. Hugenholtz P. (2010). Wrinkles in the rare biosphere: pyrosequencing errors can lead to artificial inflation of diversity estimates. *Environ. Microbiol.* 12 118–123. 10.1111/j.1462-2920.2009.02051.x 19725865

[B21] Law R. J. Bersuder P. Allchin C. R. Barry J. (2006). Levels of the flame retardants hexabromocyclododecane and tetra-bromobisphenol a in the blubber of harbor porpoises (*Phocoena phocoena*) stranded or bycaught in the UK, with evidence for an increase in HBCD concentrations in recent years. *Environ. Sci. Technol.* 40 2177–2183. 10.1021/es052416o 16646450

[B22] Li Y. N. Zhou Q. X. Wang Y. Y. Xie X. J. (2011). Fate of tetrabromobisphenol A and hexabromocyclododecane brominated flame retardants in soil and uptake by plants. *Chemosphere* 82 204–209. 10.1016/j.chemosphere.2010.10.021 21051070

[B23] Maidak B. L. Cole J. R. Lilburn T. G. Parker J. C. T. Saxman P. R. Farris R. J. (2001). The RDP-II (Ribosomal Database Project). *Nucleic Acids Res.* 29 173–174. 10.1093/nar/29.1.173 11125082 PMC29785

[B24] Morris S. Allchin C. R. Zegers B. N. Haftka J. J. H. Boon J. P. Belpaire C. (2004). Distribution and fate of HBCD and TBBP-A flame retardants in North Sea estuaries and aquatic food webs. *Environ. Sci. Technol.* 38 5497–5504. 10.1021/es049640i 15575264

[B25] Nakajima K. T. Shigeno A. Y. Nomura N. Onuma F. Nakahara T. (1999). Microbial degradation of polyurethane, polyester polyurethanes and polyether polyurethanes. *Appl. Microbiol. Biotechnol.* 51 134–140. 10.1007/s002530051373 10091317

[B26] Palace V. P. Pleskach K. Halldorson T. Danell R. Wautier K. Evans B. (2008). Biotransformation enzymes and thyroid axis disruption in juvenile rainbow trout (*Oncorhynchus mykiss*) exposed to hexabromocyclododecane diastereoisomers. *Environ. Sci. Technol.* 42 1967–1972. 10.1021/es702565h 18409622

[B27] Peng X. X. Jia X. S. (2013). Optimization of parameters for anaerobic co-metabolic degradation of TBBPA. *Bioresour. Technol.* 148 386–393. 10.1016/j.biortech.2013.08.137 24063822

[B28] Peng X. X. Qu X. D. Luo W. S. Jia X. S. (2014). Co-metabolic degradation of tetrabromobisphenol A by novel strains of *Pseudomonas* sp. and *Streptococcus* sp. *Bioresour. Technol.* 169 271–276. 10.1016/j.biortech.2014.07.002 25062538

[B29] Peng X. X. Zhang Z. L. Luo W. S. Jia X. S. (2013). Biodegradation of tetrabromobisphenol A by a novel *Comamonas* sp. strain, JXS-2-02, isolated from anaerobic sludge. *Bioresour. Technol.* 128 173–179. 10.1016/j.biortech.2012.10.051 23201509

[B30] Peng X. X. Zhang Z. L. Zhao Z. L. Jia X. S. (2012). 16S ribosomal DNA clone libraries to reveal bacterial diversity in anaerobic reactor-degraded tetrabromobisphenol A. *Bioresour. Technol.* 112 75–82. 10.1016/j.biortech.2012.02.060 22420989

[B31] Stapleton H. M. Allen J. G. Kelly S. M. Konstantinov A. Klosterhaus S. Watkins D. (2008). Alternate and new brominated flame retardants detected in U.S. house dust. *Environ. Sci. Technol.* 42 6910–6916. 10.1021/es801070p 18853808

[B32] Thomsen C. Knutsen H. K. Liane V. H. Frøshaug M. Kvalem H. E. Haugen M. (2008). Consumption of fish from a contam-inated lake strongly affects the concentrations of polybro-minated diphenyl ethers and hexabromocyclododecane in serum. *Mol. Nutr. Food Res.* 52 228–237. 10.1002/mnfr.200700123 18186101

[B33] Urakawa H. Habbena W. M. Stahl D. A. (2010). High abundance of ammonia-oxidizing *Archaea* in coastal waters, determined using a modified DNA extraction method. *Appl. Environ. Microbiol.* 76 2129–2135. 10.1128/AEM.02692-09 20118363 PMC2849251

[B34] Wang S. Q. He J. Z. (2013). Dechlorination of commercial PCBs and multiple other halogenated compounds by a sediment-free culture containing *Dehalococcoides* and *Dehalobacter*. *Environ. Sci. Technol.* 47 10526–10534. 23964900 10.1021/es4017624

[B35] Wang S. Q. Chng K. R. Wilm A. Zhao S. Y. Nagarajan N. He J. Z. (2014). Genomic characterization of three unique *Dehalococcoides* that respire on persistent polychlorinated biphenyls. *Proc. Natl. Acad. Sci. U.S.A.* 111 12103–12108. 10.1073/pnas.1404845111 25028492 PMC4142991

[B36] Wang S. Q. Chng K. R. Wu C. Bedard D. L. He J. Z. (2015). Genomic characterization of *Dehalococcoides mccartyi* strain JNA that reductively dechlorinate perchloroethene and polychlorinated biphenyls. *Environ. Sci. Technol.* 49 14319–14325. 10.1021/acs.est.5b01979 26551549

[B37] Xiao Y. Roberts D. J. (2013). Kinetics analysis of a salt-tolerant perchlorate-reducing bacterium: effects of sodium, magnesium, and nitrate. *Environ. Sci. Technol.* 47 8666–8673. 10.1021/es400835t 23789987

[B38] Yamada T. Takahama Y. Yamada Y. (2009). Isolation of pseudomonas sp strain hb01 which degrades the persistent brominated flame retardant gamma-hexabromocyclododecane. *Biosci. Biotechnol. Biochem.* 73 1674–1678. 10.1271/bbb.90104 19584526

[B39] Zhang Z. Z. Gai L. X. Hou Z. W. Yang C. Y. Ma C. Q. Wang Z. G. (2010). Characterization and biotechnological potential of petroleum-degrading bacteria isolated from oil-contaminated soils. *Bioresour. Technol.* 101 8452–8456. 10.1016/j.biortech.2010.05.060 20573503

[B40] Zhang T. Shao M. F. Ye L. (2011). 454 Pyrosequencing reveals bacterial diversity of activated sludge from 14 sewage treatment plants. *ISME J.* 6 1137–1147. 10.1038/ismej.2011.188 22170428 PMC3358032

[B41] Zubkov M. V. Fuchs B. M. Eilers H. Burkill P. H. Amann R. (1999). Determination of total protein content of bacterial cells by SYPRO staining and flow cytometry. *Appl. Environ. Microbiol.* 65 3251–3257. 10388732 10.1128/aem.65.7.3251-3257.1999 PMC91485

