# Supplementary material for: Debromination of Hexabromocyclododecane by Anaerobic Consortium and Characterization of Functional Bacteria
Source: Front Microbiol. 2018 Jul 10;9:1515. doi: 10.3389/fmicb.2018.01515 (PMC6048218; doi:10.3389/fmicb.2018.01515)
Supplement: Supplementary file 1 [file Presentation_1.PDF]

Debromination of hexabromocyclododecane by anaerobic consortium and identification of functional bacteria

Xingxing Peng[1], *Dongyang Wei*[2], *Qiyuan Huang*[1], *Xiaoshan Jia*[1*]

[1] School of environmental science and engineering, Sun Yat-sen University, Guangzhou 510275, China

[2] South China Institute of Environmental Sciences, Guangzhou 510655, China

[*] Corresponding author. E-mail: eesjxs@mail.sysu.edu.cn. Tel: +86-20-39953107

Table S1 OTUs of phylum containing dehalogenating bacteria after enrichment

phylum	genus	species	Number of OTU
	Anaerolinea	/	OTU4
Chloroflexi	/	/	OTU10
	Longilinea		OTU18
Firmicutes	*Bacillus*	/	OTU35
	Azospira	*Oryzae*	OTU2
	Enterobacter	/	OTU1
	Rhodocyclus	/	OTU58
	Dechloromonas	/	OTU12
	Zoogloea	/	OTU11
Proteobacteria	*Achromobacter*	*Insolitus*	OTU39
	Achromobacter	/	OTU17
	Acidovorax	/	OTU49
	Comamonas	*Terrigena*	OTU31
	Klebsiella	/	OTU20
	Pantoea	*Agglomerans*	OTU8
	Caulobacter	/	OTU12
	Xanthobacter	*Flavus*	OTU5

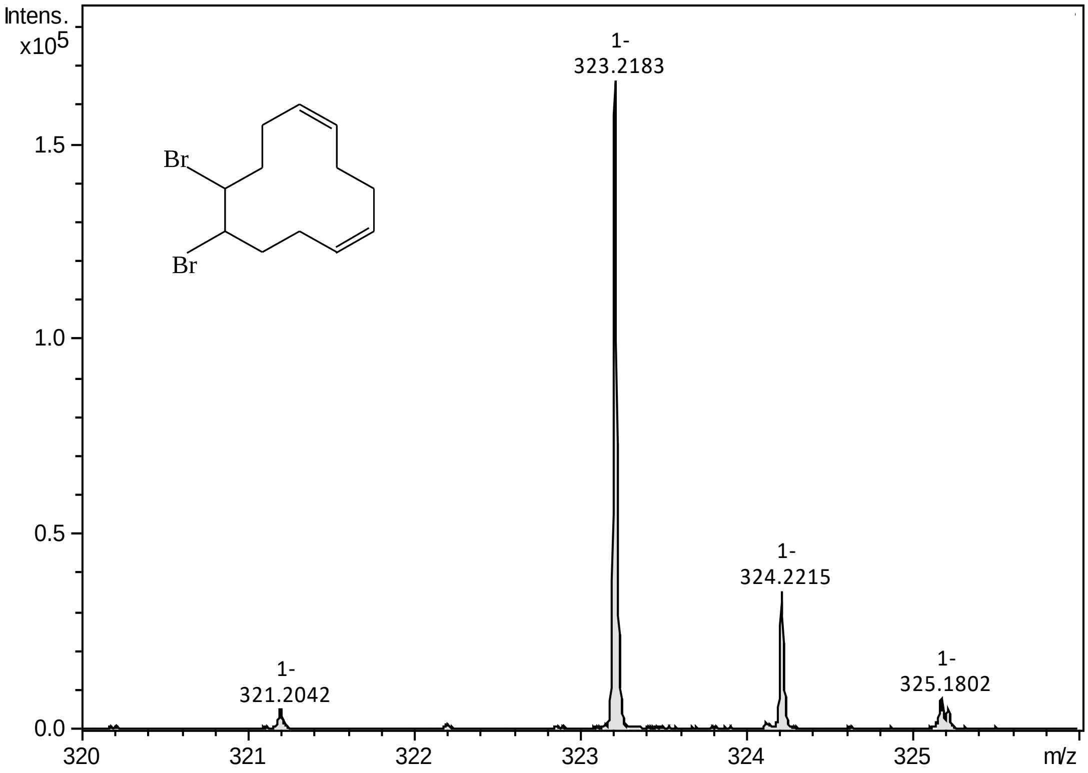

Figure S1. Mass spectrogram of dibromocyclododecadiene (DBCD)
